# Efficiency in COVID-19 inpatient care: findings from public hospitals in Iran

**DOI:** 10.1186/s13561-025-00696-7

**Published:** 2025-11-24

**Authors:** Rajabali Daroudi, Behzad Raei, Reza Goudarzi, Soheila Damiri, Hossein Ranjbaran, Zahra Shahali

**Affiliations:** 1 Department of Health Management, Policy and Economics, School of Public Health, Tehran University of Medical Sciences, Poursina Ave., Tehran, Iran; 2 Social Determinants of Health Research Center, Health and Metabolic Diseases Research Institute, Zanjan University of Medical Sciences, Zanjan, Iran; 3 Department of Public Health, School of Public Health, Zanjan University of Medical Sciences, Zanjan, Iran; 4 Health Services Management Research Center, Institute for Futures Studies in Health, Kerman University of Medical Sciences, Kerman, Iran; 5 Immunogenetics Research Center, Faculty of Medicine, Mazandaran University of Medical Sciences, Sari, Iran; 6 National Center for Health Insurance Research, Tehran, Iran

**Keywords:** Health expenditures, Cost savings, Health resources, COVID-19, Efficiency, Iran

## Abstract

**Background:**

Improving efficiency is one of the high-potential options for expanding fiscal space for health. During the pandemic, as health systems’ financial challenges intensify, the importance of utilizing resources efficiently also increases. Therefore, this study was conducted to estimate the efficiency of Iran’s public hospitals in treating COVID-19 inpatient cases.

**Methods:**

This descriptive-analytical study was based on administrative claims data from the Iran Health Insurance Organization and included 439,327 COVID-19 inpatient cases across 493 public hospitals in 2021. Epidemic waves were first identified using time-series data on daily admissions, and each patient was assigned to the corresponding wave. A logistic regression model was then fitted to estimate the probability of death based on age, sex, ICU admission, and epidemic wave. From this, a risk-adjusted survival variable (RA_surv) was calculated at the individual level and subsequently aggregated at the hospital level. Hospital efficiency was assessed using input-oriented data envelopment analysis (DEA) under variable returns to scale, with total inpatient billed charges for COVID-19 as the input and RA_surv as the output. Finally, to account for structural and contextual differences, DEA efficiency scores were adjusted using a fractional logit regression model that incorporated teaching status, specialty type, hospital size, and province fixed effects.

**Results:**

The mean of per-patient charge for COVID-19 treatment was estimated at USD 236.46 (SD = 234.48; median = 185.73), and the mean daily hospital charge was USD 46.34 (SD = 26.41; median = 41.62). These figures varied considerably across provinces, with the highest per-patient charge observed in Tehran (USD 364.98) and the lowest in South Khorasan (USD 171.37). Overall hospital efficiency scores before contextual factors adjustment ranged from 0.083 to 1.00. After adjustment, the national mean remained 0.49, although the distribution and ranking of hospitals shifted. A strong positive correlation was found between non-adjusted and adjusted efficiency scores (Spearman’s rho = 0.707, *p* < 0.001).

**Conclusion:**

This study indicated significant variation in hospital charges in COVID-19 inpatient bills in Iran’s public hospitals, and there was a relatively significant potential to save resources during the financial difficulties of Iran’s health system during the pandemic. Adopting appropriate strategies to reduce variation in clinical practice, for example, promoting the use of clinical guidelines, can significantly help reduce variation in hospital charges and subsequently improve the system’s efficiency.

**Supplementary Information:**

The online version contains supplementary material available at 10.1186/s13561-025-00696-7.

## Introduction

Efficiency is one of the mechanisms for expanding the fiscal space for health because if the same level of outputs can be produced with lower levels of inputs, part of the freed resources can be reallocated within the open health system [1]. The waste of resources in health care systems, amounting to more than one trillion dollars per year, leads to a loss of efficiency, productivity, and wealth generated in other sectors of the economy [2]. Studies show that the increase in the costs of health systems can be partly due to the inefficient use of resources [3]. So, by reducing the waste of resources such as money, manpower, buildings, and equipment, available resources can be used to provide more services or expand access and improve the quality of hospital services [4, 5].

The COVID-19 pandemic has brought about significant social and economic changes worldwide. The impact of the health sector has been greater than that of other sectors, so the system now includes new actors, priorities, interventions, and amplified socio-economic challenges. Its financial and political perspectives have also changed [6]. Estimates indicated that the COVID-19 pandemic imposed a heavy financial burden on hospitals and health systems [7]. For example, the American Hospital Association has estimated the financial impact of this epidemic on hospitals and the American healthcare system in terms of lost income at $202.6 billion (an average of $50.7 billion per month). In addition, the cost of providing an effective healthcare response to COVID-19 in low- and middle-income countries is estimated at $52 billion (US) every four weeks [8]. Following the created financial needs, governments were forced to mobilize resources for the health sector from budgetary sources. Estimates show that in 2020, the additional budget al.located to European health systems ranged from about 8% of the 2018 health budget in high-income countries to 13% in upper-middle-income countries and 23% in lower-middle-income countries [9].

During the COVID-19 era, several studies with different study designs investigated the efficiency of health systems in responding to this shock at the micro and macro levels. For example, Chia-Yu Su et al. evaluated the comparative performance of 23 countries in reducing the transmission of COVID-19 using the Data Envelopment Analysis (DEA) method [10]. Selamzade et al. employed data DEA with multi-criteria decision-making methods to assess the efficiency of OECD health systems in responding to the COVID-19 pandemic. The study revealed that countries such as Colombia, Denmark, and New Zealand demonstrated higher efficiency, whereas Slovenia and Hungary ranked among the least efficient performers [11]. Kuzior et al. applied frontier-DEA to assess public health system efficiency and COVID-19 resilience across 22 countries, finding Beveridge-type systems highly efficient and resilient, while market-based models exhibited the weakest performance [12]. Breitenbach et al. also analyzed the effectiveness of 36 countries in stabilizing infection prevalence rates and minimizing mortality rates with the DEA [13]. C.O. Henriques in Portugal [14], Fabrizio Pecoraro in Germany, France, Italy, and Spain [15], Mishra in India [16], and Sülkü in Turkey [17] evaluated the efficiency at the hospital level. Overall, these studies consistently employed DEA-based approaches at both national and hospital levels, highlighting cross-country variations in efficiency of health systems during the COVID-19 pandemic.

Due to sanctions and other socio-economic challenges, Iran was affected by the COVID-19 pandemic more than other countries in the Middle East and North Africa (MENA) [18]. According to the provided estimates in 2020 and 2021, about 1,603 and 2,324 years of life per 100,000 people were lost due to mortality and morbidity caused by COVID-19 in Iran [19]. Several estimates of the average cost of COVID-19 infected cases treatment in Iran have been reported in recent years, for example, 59,203,409 IR Rials per patient direct medical costs in 2020 [20], 33,121,029 IR Rials [21], 1434 USD [22] and $1228, and $1228 (51,578,373 IR Rials) per hospitalized patient [23], etc. However, no national-level study has compared the costs of treating COVID-19 patients across hospitals or examined the efficiency of such expenditures. This study aims to address this gap by analyzing hospitalization data from public hospitals in Iran in 2021.

## Methods

In this study, we estimated the efficiency of Iranian public hospitals in managing COVID-19 inpatients in 2021 using the DEA method and subsequently calculated each hospital’s potential cost-saving capacity based on the adjusted efficiency scores. Efficiency and cost-saving capacity were first computed at the hospital level and then aggregated to the provincial level to provide a summarized view of regional variations.

### Data and study population

In 2021, Iran had about 1030 hospitals (with 161,377 beds), and 68% of them were public hospitals (with 73.9% of total hospital beds) [24]. Public hospitals are primarily financed by basic health insurance organizations, including the Social Security Organization (SSO), the Iran Health Insurance Organization (IHIO), and the Armed Forces Medical Service Insurance Organization (AFMSIO) [19, 25]. These organizations have established claims assessment mechanisms to ensure billing compliance. Unlike other insurers that still rely on paper-based claim processing, the IHIO uses a computerized system linked to hospital information systems (HIS), ensuring accurate financial data that can be effectively used for policymaking [26]. We used stored patient demographic characteristics and financial data in the IHIO electronic claim processing system for patients admitted to public hospitals in 2021 due to COVID-19. Patients were identified using ICD-10 codes U07.1 (laboratory-confirmed) and U07.2 (clinically/epidemiologically diagnosed, but without laboratory confirmation), in accordance with the Iranian Ministry of Health guidelines [27]. These codes are regularly monitored by insurance auditors, strengthening their reliability despite the possibility of some misclassification.

The analytic sample comprised all inpatients hospitalized for at least one day with a primary diagnosis of COVID-19, while outpatient visits were excluded. Hospitals with minimal COVID-19 caseloads (total length of stay < 500 patient-days) were also excluded to enable meaningful comparisons. The dataset included hospital identifiers, patient demographics, clinical information, and detailed financial data (consultations, medicines, consumables, room and board, laboratory tests, imaging, etc.).

### Efficiency measurement

In this study, each hospital was considered a decision-making unit (DMU), and efficiency in managing COVID-19 inpatients was evaluated through four steps: identifying epidemic waves, risk-adjusting survival, measuring efficiency using DEA, and adjusting efficiency scores based on hospital contextual factors.

Efficiency was assessed from the payer’s perspective. Unlike operational efficiency studies that focus on staff, beds, or equipment, this approach is particularly relevant in contexts where expenditure control is a key policy concern and financial data are the most reliable source of information. An input-oriented DEA model under the variable returns-to-scale (VRS) assumption was employed [28, 29]. Total inpatient COVID-19 expenditures were used as the input, and risk-adjusted survival (RA_surv) was estimated via logistic regression, controlling for age, sex, ICU admission, and epidemic wave, which were considered as the output. This specification was designed to capture hospitals’ ability to minimize costs while maintaining patient survival outcomes.

### Patient-level risk adjustment

To account for patient case-mix heterogeneity and construct the DEA output variable, risk-adjusted survival was estimated using a logistic regression model. In efficiency analyses, risk-adjusted health outcomes, particularly mortality rates, are commonly treated as undesirable outputs in DEA models. For example, Clement et al. [30] used logistic regression to estimate risk-adjusted mortality and incorporated it with service volumes, while Bilsel and Davutyan [31] applied a similar adjustment in evaluating hospital efficiency in Turkey. In our study, however, we adopted a conceptually equivalent but more intuitive measure: risk-adjusted survival. At the individual level, the following model was fitted:

$$\begin{aligned}\:\text{l}\text{o}\text{g}\text{i}\text{t}\left(\text{P}\left({\text{d}\text{e}\text{a}\text{t}\text{h}}_{\text{i}\text{}}\right)\right) & ={\beta\:}_{0}+{\beta\:}_{1}\left({\text{a}\text{g}\text{e}\:\text{g}\text{r}\text{o}\text{u}\text{p}}_{i}\right)+{\beta\:}_{2}\left({\text{s}\text{e}\text{x}}_{i}\right)\\ & +{\beta\:}_{3}\left({\text{I}\text{C}\text{U}\:\text{a}\text{d}\text{m}\text{i}\text{s}\text{s}\text{i}\text{o}\text{n}}_{i}\right)+{\beta\:}_{4}\left({\text{w}\text{a}\text{v}\text{e}}_{i}\right)\end{aligned}$$

Epidemic waves were identified using smoothed daily admission counts (7-day moving average), and each admission was assigned to a wave category (waves 1–3, with non-wave as reference).

Predicted probabilities of death ($p\hat{}_i$) were calculated for each patient. Risk-adjusted survival at the patient level was then defined as:

$${\mathrm R{\mathrm A}_-\mathrm{su}\mathrm r\mathrm v}_i=1-\mathrm p{\widehat{}}_i\;$$

and then aggregated at the hospital level:

$$\:{RA\_surv}_{h}={\sum\:}_{i\in\:\text{h}}{RA\_surv}_{i}$$

Thus, $\:{RA\_surv}_h$ reflects the expected number of survivors adjusted for age, sex, ICU admission, and epidemic wave.

Defining a COVID-19 wave is methodologically complex and subject to debate, as different criteria can lead to varying interpretations [32]. While a comprehensive epidemiological exploration of wave dynamics was not the primary aim of this study, we acknowledged these challenges and applied a clear, scientifically grounded, and transparent approach to identify epidemic waves in our dataset. This ensured that our analysis remained methodologically sound without diverging from the specific focus of the study. To delineate distinct epidemic waves, we applied a multi-step time-series analysis of daily hospital admissions for COVID-19. First, daily counts (𝑥_𝑡_) were smoothed using a 7-day centered moving average to reduce short-term fluctuations such as weekday–weekend variations:

$$\begin{aligned} & \:{MA7}_{t}=\\ & \frac{{x}_{t-3}+{x}_{t-2}+{x}_{t-1}+{x}_{t}+{x}_{t+1}+{x}_{t+2}+{x}_{t+3}}{7}\end{aligned}$$

Epidemic peaks were defined as local maxima where:

$$\:{MA7}_{t+1}<{MA7}_{t}\:\& \:\:{MA7}_{t-1}<{MA7}_{t}\:$$

while troughs were defined as local minima where: $\:{MA7}_{t+1}>{MA7}_{t}\:\& \:\:{MA7}_{t-1}>{MA7}_{t}\:$

Each wave was defined from the trough before a peak, through the peak, to the next trough. To keep only meaningful peaks, we applied two filters: at least 20 days between peaks to avoid short-term fluctuations, and a prominence threshold to distinguish peaks from neighboring troughs. The 20-day gap was a conservative empirical choice. Similar approaches using moving averages and local extrema have been reported previously [32, 33].

### Contextual adjustment of efficiency scores

To isolate hospital-level contextual influences from the DEA efficiency scores and provide policy-relevant, size-aware comparisons, we modeled the hospital efficiency score 𝜃_ℎ_ ∈ [0,1] using a fractional logit regression (Papke–Wooldridge). This specification is appropriate for bounded outcomes and accommodates the few boundary observations at unity. The model related efficiency to structural characteristics that were not used in the risk adjustment of survival (to avoid double-adjustment), namely teaching status, specialty profile, hospital size, and province fixed effects:

$$\begin{aligned}E (\theta_{h} | Z_{h}) & = \text{logit}^{-1} (\beta_{0}+ \beta_{1} Teaching_{h} \\ & + \beta_{2}SingleSpecialty_{h} +\beta_{3}\! \ln(Beds_{h}) \\ & + \gamma_{Province(h)}), \quad w_{h} = CaseVolume_{h}\end{aligned}$$

Because hospitals treated very different numbers of COVID-19 patients, regressions were estimated with probability weights equal to each hospital’s COVID-19 admission volume (i.e., pweights = case volume), so that estimates reflect the relative importance of higher-volume hospitals. ICU admission and epidemic wave indicators were not included at this stage because they had already been incorporated upstream in the patient-level logistic model used to form the risk-adjusted survival output for DEA; re-including them would constitute post-treatment adjustment of the DEA outcome.

From the fitted model we obtained fitted values $\:\widehat{{\theta\:}_{h}}$. To remove the portion of efficiency associated with contextual factors while preserving the overall scale, we centered fitted values and subtracted them from observed scores:

$$\:{\theta\:}_{h}^{adj}={\theta\:}_{h}-({\widehat{\theta\:}}_{h}-\stackrel{-}{\widehat{\theta\:}})$$

where $\:\stackrel{-}{\widehat{\theta\:}}$ is the sample mean of $\:{\widehat{\theta\:}}_{h}$​. This yields context-adjusted efficiency scores that are interpretable on the original 0–1 scale.

### Estimating cost-saving capacity

For each hospital, the potential cost savings were then computed using the following equation. In this equation, the $\:{\theta\:}_{h}^{adj}$ stands for context-adjusted efficiency scores.

$$\:{Cost\:Saving\:capacity}_{h}={\text{T}\text{o}\text{t}\text{a}\text{l}\:\text{C}\text{o}\text{s}\text{t}}_{h}\times\:(1-{\theta\:}_{h}^{adj})$$

## Results

### Unit cost analysis

This study evaluated data on hospital charges from 439,179 COVID-19-hospitalized patients from 493 Iranian public hospitals (Additional details are available in Additional file 1). Billed amounts paid for inpatient services reached nearly $103,870,687. Estimated charges per patient were $236.46 (Std. Dev: $234.48, median: $185.73). This figure varies among different regions (as grouped by province), ranging from $364.98 in Tehran to $267.46 in Alborz Province, $266.63 in Isfahan Province, $265.66 in Khuzestan Province, and $263.84 in Golestan Province, with a low of $171.37 in South Khorasan Province. The estimated charge for each day of a hospital stay is approximately $46.34 (Standard Deviation: $26.41, Median: $41.62). This figure also differed significantly across provinces, ranging from as high as $64.14 in Sistan & Baluchistan Province, $59.62 in Khuzestan Province, and $59.59 in Tehran Province, to as low as $40.27 in Ardabil Province and $39.87 in Lorestan Province. Generally, the main charged groups were medicine and medical supplies (42.00%), followed by hoteling (28.84%), and then visits and consultation (14.21%) (Table 1).

**Table 1:** Descriptive statistics of COVID-19 inpatient hospital charges

Province	Total Charge (USD)	Per-patient charges (USD)			Per-day charges (USD)			The contribution of different service groups to the total charge											
		Mean	SD	Median	Mean	SD	Median	visit		hoteling		Medicine & medical supplies		imaging		laboratory		Other	
								Mean	SD	Mean	SD	Mean	SD	Mean	SD	Mean	SD	Mean	SD
East Azerbaijan	5,044,805	241.6	241.9	189.5	42.4	20.6	38.5	14.1	7.6	31.2	14.3	41.4	20.9	2.3	3.4	8.8	6.9	2.3	8.1
West Azerbaijan	5,937,673	211.8	174.6	174.9	41.8	18.6	38.8	12.9	7.0	29.9	14.0	48.5	20.1	2.0	3.2	5.8	4.5	0.9	5.7
Ardabil	1,887,112	204.5	201.6	168.9	40.3	18.0	37.9	13.4	7.2	28.9	12.8	44.0	21.8	3.1	5.1	8.6	6.6	2.1	8.2
Isfahan	7,369,555	266.6	280.4	197.4	44.3	22.3	40.6	15.2	8.0	31.6	14.0	35.2	21.1	4.5	6.1	10.4	7.1	3.2	9.3
Alborz	2,143,455	267.5	223.8	214.5	51.6	30.8	44.2	12.8	6.7	29.2	13.3	46.1	18.4	2.1	3.3	7.5	5.0	2.3	8.0
Ilam	1,099,100	179.2	139.9	153.6	45.5	19.2	42.9	15.1	8.7	25.1	12.2	50.3	19.6	2.1	3.4	5.7	5.2	1.8	6.3
Bushehr	915,521	197.6	177.2	162.8	43.0	21.4	39.5	17.7	9.1	27.4	12.7	41.4	21.0	2.9	3.9	7.2	6.0	3.4	8.6
Tehran	12,000,000	365.0	373.4	256.2	59.6	42.0	51.2	13.6	7.6	30.4	14.9	35.6	20.0	3.7	5.3	11.4	7.2	5.4	12.5
ChaharM& Bakhtiari	1,858,093	244.2	250.9	188.2	47.4	23.2	43.7	17.4	9.4	26.3	11.0	34.9	21.9	4.8	5.7	14.2	8.1	2.5	8.1
South Khorasan	1,436,978	171.4	164.7	140.1	42.7	21.2	39.8	16.5	8.3	27.6	12.7	38.0	22.5	4.5	5.6	11.8	8.5	1.6	6.8
Razavi Khorasan	7,648,664	229.4	240.0	172.3	42.8	21.9	38.7	13.7	8.1	32.1	14.6	38.3	21.1	4.1	4.8	8.5	6.2	3.2	9.7
North Khorasan	1,904,478	186.0	145.2	166.4	44.0	21.6	40.8	13.4	7.2	26.6	12.0	43.5	22.0	4.0	5.0	9.7	8.3	2.9	11.4
Khuzestan	5,884,560	265.7	264.5	194.5	59.6	42.6	48.1	15.1	7.6	28.3	16.5	44.7	21.5	2.9	4.1	5.9	4.0	3.1	10.3
Zanjan	1,737,208	250.1	198.6	208.4	47.0	23.0	43.5	13.4	7.3	27.3	12.7	45.8	19.9	2.0	3.1	8.4	5.7	3.1	6.1
Semnan	1,041,931	243.7	207.0	199.0	45.7	22.6	42.2	11.8	7.0	29.5	13.1	43.1	22.4	3.0	4.0	8.8	6.5	3.9	9.4
Sistan & Baluchestan	1,718,682	237.4	230.9	183.4	64.1	41.4	51.3	15.2	8.9	25.5	15.4	42.2	24.1	3.6	4.7	7.9	6.6	5.6	17.2
Fars	5,820,122	220.0	214.7	178.5	45.3	21.3	42.1	14.4	7.6	27.1	12.5	42.8	21.9	3.4	5.1	9.1	6.9	3.2	8.7
Qazvin	1,518,612	233.0	246.0	170.2	42.9	23.7	38.3	16.5	7.6	31.2	13.8	35.6	21.0	3.5	4.7	11.3	7.3	1.9	8.0
Qom	1,559,689	231.2	221.2	181.4	41.7	22.7	37.6	12.8	7.4	32.1	14.3	37.4	21.8	3.9	5.1	11.2	8.1	2.7	7.2
Kurdistan	2,712,086	209.1	203.3	167.7	41.2	17.5	38.0	6.8	8.5	28.2	11.8	50.1	17.8	1.9	3.4	5.8	4.3	7.2	10.0
Kerman	5,175,387	218.5	220.2	171.7	45.8	24.3	41.7	15.7	8.6	27.6	13.1	37.7	22.7	4.3	5.0	10.7	7.5	4.1	12.7
Kermanshah	3,041,775	217.5	191.6	182.5	43.8	20.8	40.1	14.5	8.6	28.0	13.4	45.4	19.6	3.1	4.2	7.2	5.6	1.8	6.9
Kohgiluyeh & BoyerA	1,490,249	219.8	204.4	179.1	42.8	19.0	40.3	21.2	8.3	28.6	12.3	37.2	21.1	2.1	3.9	9.2	7.5	1.8	5.8
Golestan	3,101,441	263.8	235.8	209.5	48.1	25.0	44.1	14.1	8.0	27.6	13.0	44.6	20.6	3.0	3.8	8.6	6.1	2.1	8.6
Gilan	2,027,770	199.2	186.3	161.8	41.3	20.2	38.1	13.5	7.6	27.2	13.2	42.1	23.2	3.2	4.4	11.8	8.2	2.4	8.1
Lorestan	3,228,390	182.6	144.6	166.5	39.9	18.9	37.9	13.9	7.7	27.6	12.7	45.3	22.6	2.8	4.5	9.1	7.8	1.3	6.2
Mazandaran	4,585,425	243.3	205.9	206.2	47.1	26.4	43.0	14.2	7.8	29.0	14.1	44.1	22.2	3.0	4.4	7.8	5.4	2.0	8.5
Markazi	1,489,480	223.0	197.7	182.3	45.6	24.3	41.3	14.0	7.8	28.5	13.4	44.1	21.1	2.7	4.1	8.7	7.5	2.0	5.9
Hormoz Gan	2,713,674	196.7	184.1	166.2	48.2	23.4	44.9	15.8	8.9	25.3	12.5	42.6	22.5	3.6	4.2	9.9	8.1	2.9	10.7
Hamadan	4,195,932	225.2	174.1	194.1	42.3	18.3	40.0	13.6	7.1	28.4	12.6	46.9	20.1	2.3	3.6	7.7	5.4	1.1	6.1
Yazd	1,582,840	229.9	210.2	194.3	48.2	22.5	44.9	15.7	9.1	26.1	11.7	45.9	21.1	3.0	4.1	7.7	6.6	1.7	5.8
Total	103,870,687	236.5	234.5	185.7	46.3	26.4	41.6	14.2	8.1	28.8	13.7	42.0	21.7	3.2	4.6	8.9	6.9	2.9	9.4

**Fig. 1:**
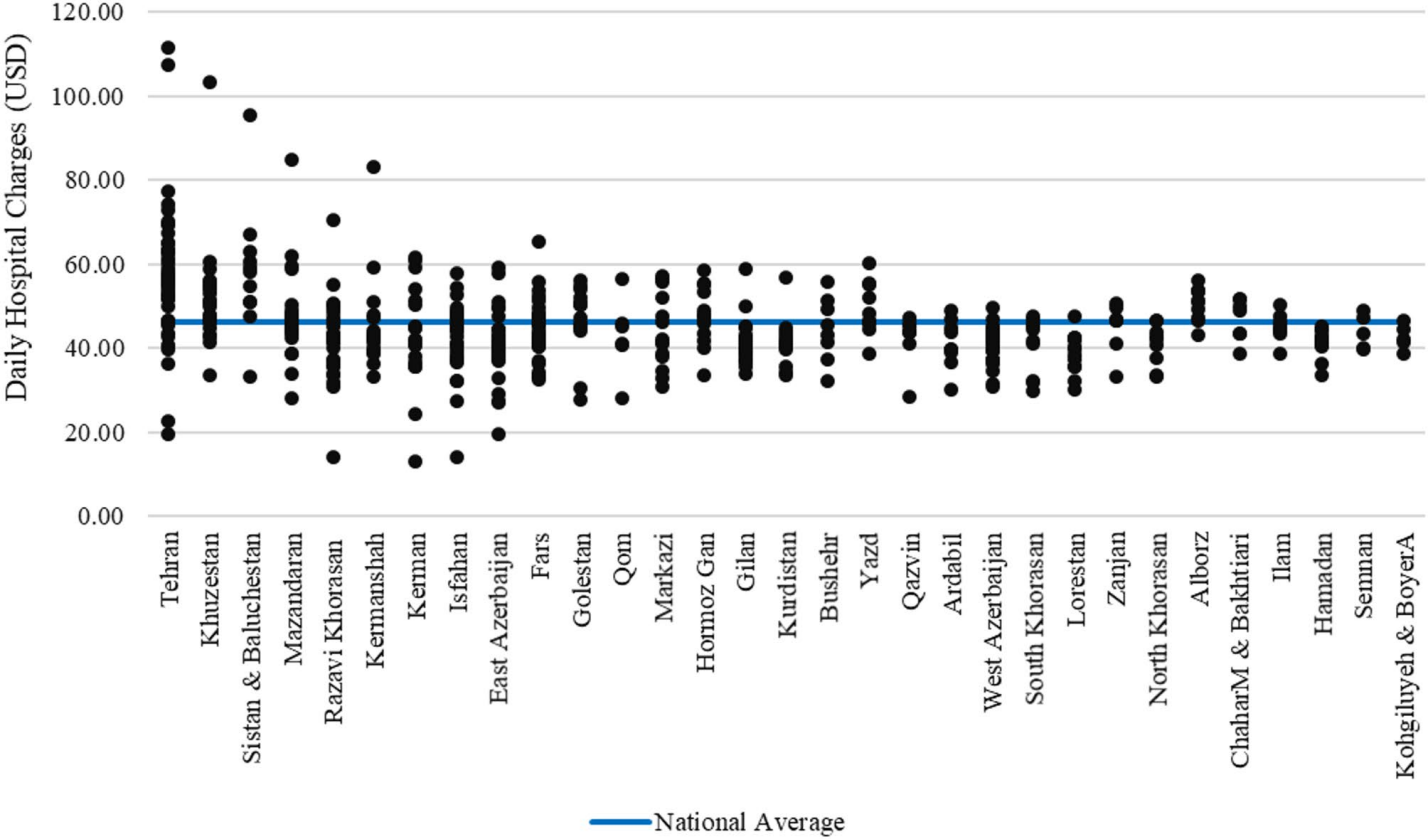
Average daily hospital charges across hospitals by province

Figure 1 illustrates the variation of per-day charges across hospitals for each province. Analysis reveals that although difference was found in every province, the range of such differences considerably differed across provinces. In Tehran province, for example, the per-day charges ranged widely from $19.4 to $111.6, thereby highlighting a significant difference across hospitals. Likewise, Khuzestan province had a range between $33.6 and $103.5, whereas Sistan & Baluchistan had fluctuations between $33.2 and $95.6. In contrast, Semnan province and Kohgiluyeh & Boyer Ahmad province had the lowest ranges, with a difference in their respective daily charges of merely $9.3 and $7.9, respectively. Compared to national average figures, provinces such as Tehran not only reflected the highest range difference across hospitals but also had their average daily charges considerably elevated compared to national average figures. In contrast, provinces such as Semnan had a trend towards reporting below national average values (Fig. 1).

The results of the logistic regression model used to estimate patient-level mortality probabilities, which formed the basis for calculating risk-adjusted survival, are presented in Supplementary Table S2. The probability of death was lower during the first wave (OR = 0.94, 95% CI: 0.91–0.97), significantly higher during the second wave (OR = 1.14, 95% CI: 1.11–1.18), and lower again during the third wave (OR = 0.75, 95% CI: 0.72–0.78), compared with periods outside the epidemic waves. Age was strongly associated with increased mortality. Patients aged 21–50 years had nearly twice the odds of death compared with those younger than 20 years (OR = 1.91, 95% CI: 1.76–2.08), and patients older than 50 years had more than sixfold higher odds (OR = 6.48, 95% CI: 5.99–7.01). Female patients had a lower death probability than male patients, with an odds ratio of 0.79 (95% CI: 0.77–0.81). ICU admission emerged as the strongest predictor, with patients admitted to intensive care having more than 15-fold higher odds of death (OR = 15.1, 95% CI: 14.8–15.5).

**Fig. 2:**
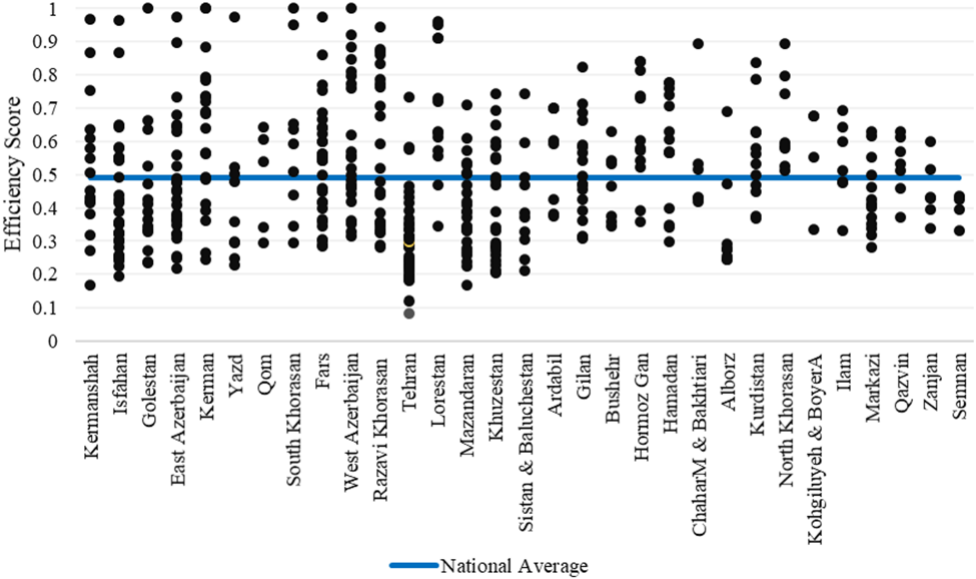
Distribution of hospital efficiency scores across provinces (before adjustment for contextual variables)

The analysis of hospital efficiency scores in the baseline model (before adjustment for contextual factors) indicates that overall scores across the country ranged from 0.083 to 1.00. Figure 2 presents the estimated efficiency scores of all hospitals, categorized by province. As shown, Kermanshah province (range: 0.167–0.968) exhibited the widest variation in hospital efficiency scores, whereas Semnan province (range: 0.331–0.434) showed the narrowest variation. Full efficiency scores (1.00) were observed in several provinces, with the highest frequency recorded in Kerman province, where three hospitals achieved the maximum score. Comparing hospital efficiency scores with the national average of 0.498 reveals that in provinces such as Tehran and Mazandaran, a larger proportion of hospitals scored below the national benchmark. In contrast, Lorestan province had the majority of its hospitals scoring above the national average. These findings highlight the substantial heterogeneity in efficiency across hospitals and provinces in Iran.

The results of the fractional logit regression, which assessed the association between hospital contextual factors and efficiency scores used to obtain the adjusted efficiency values, are presented in Supplementary Table S3. Teaching status was not significantly associated with efficiency, whereas being a single-specialty hospital was negatively associated with efficiency (β = − 0.339, *p* = 0.050; marginal effect = − 0.084, *p* = 0.053). Larger hospitals demonstrated higher efficiency, as indicated by the positive effect of ln(beds) (β = 0.158, *p* = 0.016; marginal effect = 0.038, *p* = 0.015). Substantial regional heterogeneity was observed: hospitals in West Azerbaijan, North Khorasan, Kerman, Lorestan, and Hormozgān had significantly higher efficiency compared to the reference province (East Azerbaijan), while hospitals in Tehran and Semnan had lower efficiency scores.

Table 2 compares hospital efficiency scores before and after contextual adjustment using fractional logit regression, as well as the corresponding potential cost-saving capacity. At the national level, the mean DEA efficiency score was 0.49 (SE = 0.01; median = 0.48). After contextual adjustment, the mean score remained stable (0.49), but quartile distributions shifted, indicating re-ranking of hospitals once teaching status, specialty, size, and province were accounted for. Provinces such as North Khorasan, Lorestan, and Kerman showed relatively higher non-adjusted efficiency, while Tehran, Semnan, and Mazandaran recorded lower scores. Adjustment attenuated some of these differences, suggesting that part of the variation reflected structural or regional factors rather than intrinsic performance. Potential cost-saving capacity also varied substantially: in the unadjusted model, savings were concentrated in provinces with lower raw efficiency (e.g., Tehran, Isfahan, Khuzestan), whereas adjusted estimates redistributed potential savings more evenly across regions. Overall, the adjusted results provide a more equitable basis for cross-provincial comparisons of efficiency and cost-saving opportunities.

**Table 2:** Results for efficiency measurement and cost-saving potential estimation

	Non-adjusted DEA efficiency								Adjusted efficiency (fractional logit)							
Province	Efficiency score			Hospital distribution in efficiency quartiles (%)				Potential savings (USD)	Efficiency score			Hospital distribution in efficiency quartiles (%)				Potential savings (USD)
	Mean	SD	Median	Q1	Q2	Q3	Q4		Mean	SD	Median	Q1	Q2	Q3	Q4	
East Azerbaijan	0.473	0.190	0.438	25.0	32.1	17.9	25.0	2,467,547	0.493	0.196	0.438	28.6	28.6	17.9	25.0	2,472,162
West Azerbaijan	0.59	0.201	0.542	8.0	24.0	32.0	36.0	1,812,713	0.448	0.189	0.420	48.0	16.0	4.0	32.0	2,751,736
Ardabil	0.597	0.176	0.603	0.0	33.3	22.2	44.4	514,430	0.444	0.163	0.476	33.3	22.2	22.2	22.2	858,784
Isfahan	0.424	0.180	0.356	42.4	24.2	21.2	12.1	3,945,254	0.518	0.167	0.472	15.2	36.4	21.2	27.3	3,478,060
Alborz	0.324	0.146	0.274	80.0	0.0	10.0	10.0	1,218,246	0.428	0.160	0.381	40.0	40.0	10.0	10.0	997,960
Ilam	0.514	0.125	0.496	12.5	12.5	50.0	25.0	508,064	0.520	0.129	0.493	12.5	25.0	25.0	37.5	510,359
Bushehr	0.513	0.171	0.501	0.0	50.0	25.0	25.0	421,516	0.525	0.174	0.506	12.5	25.0	50.0	12.5	435,858
Tehran	0.302	0.124	0.287	75.0	18.2	4.5	2.3	8,368,397	0.543	0.134	0.506	2.3	31.8	40.9	25.0	5,744,693
ChaharM & Bakhtiari	0.538	0.166	0.515	0.0	42.9	42.9	14.3	823,355	0.516	0.173	0.472	14.3	42.9	28.6	14.3	918,437
South Khorasan	0.599	0.231	0.583	10.0	20.0	30.0	40.0	385,791	0.417	0.239	0.399	40.0	30.0	10.0	20.0	665,781
Razavi Khorasan	0.555	0.222	0.486	19.2	26.9	15.4	38.5	2,526,604	0.440	0.207	0.369	50.0	11.5	7.7	30.8	3,651,751
North Khorasan	0.69	0.168	0.596	0.0	0.0	55.6	44.4	398,872	0.459	0.155	0.373	44.4	11.1	22.2	22.2	861,218
Khuzestan	0.399	0.173	0.338	50.0	8.3	29.2	12.5	3,018,923	0.475	0.188	0.397	41.7	12.5	16.7	29.2	2,713,244
Zanjan	0.434	0.095	0.427	14.3	57.1	28.6	0.0	881,116	0.512	0.075	0.512	0.0	28.6	57.1	14.3	788,353
Semnan	0.406	0.045	0.426	20.0	80.0	0.0	0.0	599,039	0.538	0.026	0.542	0.0	0.0	100.0	0.0	475,001
Sistan & Baluchestan	0.416	0.156	0.384	36.4	27.3	27.3	9.1	994,378	0.523	0.154	0.511	18.2	18.2	36.4	27.3	827,479
Fars	0.533	0.168	0.546	12.5	25.0	34.4	28.1	2,436,264	0.503	0.169	0.509	21.9	25.0	28.1	25.0	2,785,067
Qazvin	0.503	0.107	0.524	12.5	25.0	37.5	25.0	697,179	0.526	0.108	0.506	0.0	25.0	37.5	37.5	704,231
Qom	0.571	0.253	0.572	16.7	16.7	33.3	33.3	603,635	0.476	0.276	0.452	33.3	33.3	16.7	16.7	777,621
Kurdistan	0.546	0.148	0.534	0.0	30.8	38.5	30.8	999,945	0.478	0.125	0.490	23.1	23.1	38.5	15.4	1,246,841
Kerman	0.63	0.219	0.682	9.5	14.3	19.0	57.1	1,210,340	0.426	0.222	0.498	38.1	9.5	28.6	23.8	2,422,424
Kermanshah	0.517	0.206	0.473	17.6	29.4	23.5	29.4	1,297,801	0.461	0.213	0.452	41.2	17.6	17.6	23.5	1,498,790
Kohgiluyeh & BoyerA	0.595	0.159	0.675	20.0	0.0	20.0	60.0	512,490	0.486	0.147	0.556	20.0	20.0	40.0	20.0	677,542
Golestan	0.435	0.197	0.377	37.5	31.3	12.5	18.8	1,798,755	0.549	0.184	0.510	12.5	31.3	31.3	25.0	1,479,400
Gilan	0.502	0.149	0.472	15.8	26.3	36.8	21.1	935,557	0.520	0.156	0.505	15.8	26.3	26.3	31.6	944,516
Lorestan	0.688	0.187	0.638	0.0	7.1	21.4	71.4	671,226	0.443	0.185	0.389	35.7	28.6	7.1	28.6	1,482,337
Mazandaran	0.394	0.132	0.381	38.5	30.8	23.1	7.7	2,544,784	0.506	0.132	0.464	15.4	38.5	15.4	30.8	2,111,181
Markazi	0.443	0.111	0.419	14.3	50.0	21.4	14.3	740,975	0.496	0.096	0.484	7.1	35.7	42.9	14.3	682,359
Hormoz Gan	0.611	0.170	0.578	0.0	23.1	38.5	38.5	796,812	0.465	0.165	0.440	30.8	23.1	23.1	23.1	1,250,647
Hamadan	0.565	0.172	0.568	6.7	26.7	26.7	40.0	1,648,111	0.510	0.162	0.508	26.7	13.3	26.7	33.3	1,976,208
Yazd	0.439	0.218	0.419	40.0	10.0	40.0	10.0	816,123	0.454	0.221	0.444	40.0	30.0	20.0	10.0	815,594
Total	0.490	0.197	0.467	-	-	-	-	52,190,816	0.493	0.196	0.478	-	-	-	-	52,265,335

**Fig. 3:**
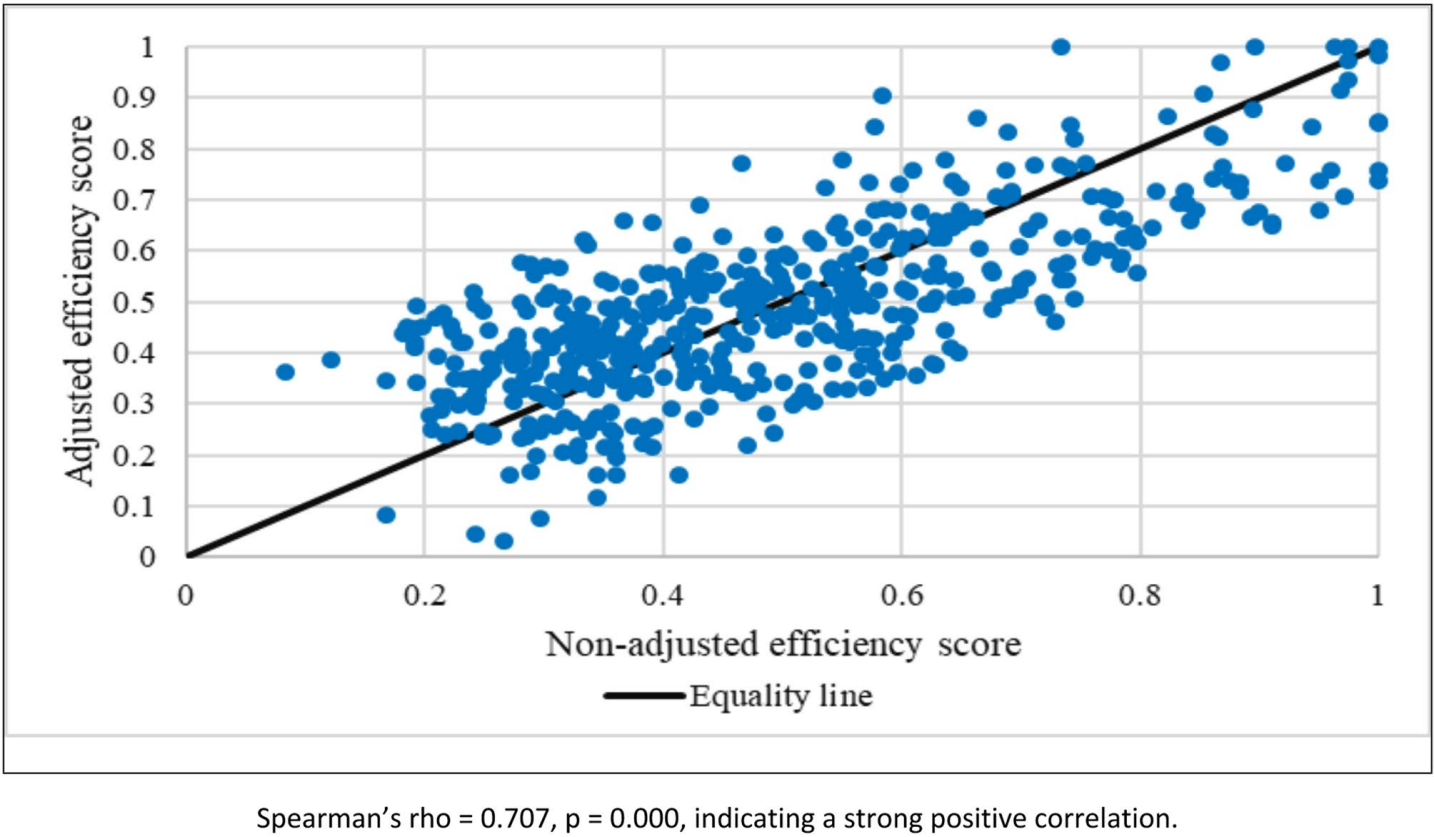
Scatter plot of adjusted and non-adjusted hospital efficiency scores

Figure 3 illustrates the scatter plot of non-adjusted versus adjusted efficiency scores across hospitals. The points cluster around the equality line (y = x), highlighting a strong association between the two measures. Nevertheless, the distribution reveals that efficiency scores increased after adjustment for about 52% of the hospitals, while approximately 47% experienced a decline. In just one case, the adjusted and non-adjusted scores were identical. The Spearman correlation between the non-adjusted efficiency score and the Adjusted efficiency score was 0.707, which was statistically significant at the 0.01 level (*p* = 0.000). This finding indicates a strong positive correlation between the two efficiency measures, such that higher non-adjusted efficiency values are associated with higher adjusted efficiency values.

## Discussion

This study aimed to assess the efficiency of public hospitals in Iran in managing hospitalized COVID-19 patients and to estimate the potential cost-saving capacity. Data from over 439,000 admissions across 493 hospitals in 2021 were analyzed, and efficiency scores were calculated using an input-oriented DEA model, with hospital charges as inputs and risk-adjusted survival as outputs. The scores were subsequently adjusted based on hospital structural characteristics, yielding a mean efficiency of 0.49.SS. Several studies have been conducted in Iran to evaluate the efficiency of hospitals using various perspectives and methodologies. For example, in Malekzadeh et al.‘s study, which employed the Pabon Lasso approach, it was demonstrated that the efficiency of units in general hospitals decreased at the onset of the epidemic [34]. Raadabadi et al.‘s study in the west of Iran [35] and Mahmoodi et al.‘s study in one of the eastern provinces of Iran, with the Pabon Lasso method, reports similar results [36]. Mirmozaffari et al. investigated the efficiency of 56 hospitals during the COVID-19 pandemic using two DEA approaches and random frontier analysis (SFA). The average scores estimated using these methods were 0.87 and 0.84, respectively. in this study, in addition to cost, they also considered other inputs such as human power, the number of hospital beds, etc [25]. Although there are considerable differences in the settings and methodological approaches of these studies, their overall findings consistently emphasize that there has been a substantial potential for improving the efficiency of Iran’s health system during the COVID-19 era.

The potential for resource savings in crises such as COVID-19 is a critical concern for health insurance organizations. In this study, instead of operational resources such as human resources, including physicians, and hospital infrastructures, hospital charges were used as the input. Despite considerable variation across hospitals, the average charge per admitted COVID-19 patient was USD 236.46 (median USD 185.73), and per hospital day USD 46.34 (median USD 41.62). Medications and consumables (42.01%), hoteling services (28.85%), and physician visits (14.21%) were the largest cost categories. In recent years, several studies have estimated hospitalization costs of COVID-19 or the broader economic burden at national or sub-national levels. Owing to differences in methodology, study setting, exchange-rate conversion, and study period, a wide range of mean costs has been reported. For example, Khandehroo et al. analyzed 1,015 hospitalized patients in a teaching hospital and found an average charge of 77,107,720 IRR (≈ USD 2,461 PPP) and a median of 42,410,477 IRR, with medications and consumables comprising nearly 37% of total costs [37]. Ramezani-Doroh et al. analyzed 909 hospitalized COVID-19 patients in Sina Hospital, Hamedan, and reported a median inpatient cost of USD 134.48 (range: 19.19–2,397.54), with higher costs observed in patients with longer stays (>9 days, median USD 507.30). Medications and consumables accounted for the largest share of expenses, comprising nearly 58% of total costs [38]. Damiri et al. analyzed data from 1,324 hospitalized COVID-19 patients at Imam Khomeini Hospital in Tehran University of Medical Sciences and found an average inpatient charge of 33,121,029 IRR (≈ USD 209.22) [21]. Haji Aghajani et al. analyzed 991 hospitalized COVID-19 patients in Imam Hossein Hospital, Tehran, and reported a median hospitalization cost of USD 271.1 (IQR 170.7–489.6). Hoteling services and medications comprised the largest share of expenditures [39].

About $104 million was charged for COVID-19 inpatient cases treatment in public hospitals. The saving potential of charges estimated before and after adjustment was $52,190,816 and $52,265,335, respectively (51.00% and 51.07% of total charges). In this study, only the hospital bills of the IHIO-insured population, who constitute almost half of the country’s population (42,158,648 from 84,055,000), have been included, so about twice this amount could be saved in the inpatient sector of Iran’s health system. Generally, the study’s results reflect the high capacity of saving financial resources available for the Iranian health system during the pandemic. The relatively low to moderate efficiency of Iran’s health system in responding to the COVID-19 pandemic has also been confirmed in other studies. For example, Breitenbach et al. conducted an input-oriented DEA of 36 countries, assessing health system efficiency in managing COVID-19 under three alternative models that differed in their outputs (recovery-to-infection ratio, recovery-to-death ratio, and total recoveries). The average technical efficiency score across countries was approximately 0.53, whereas Iran consistently exhibited lower efficiency (around 0.33) across all models, highlighting considerable potential for improving resource allocation compared with other countries [13]. Babaei-Meybodi et al. evaluated the efficiency of 50 countries in managing COVID-19 during April–June 2020 using an input-oriented, two-stage DEA BCC model with Variable Returns to Scale. The first stage assessed outbreak control (inputs: population, number of tests, outbreak duration; output: confirmed cases), while the second examined treatment (inputs: confirmed cases, health expenditure; outputs: recoveries and deaths). Mean efficiency scores across countries varied by period, averaging 0.5152, 0.4423, and 0.5210 for Stage 1, and 0.4900, 0.3500, and 0.4700 for Stage 2. Iran ranked 10th overall with an average efficiency of 0.6592, scoring 1.0000, 0.0583, and 0.0641 in Stage 1 and 0.9443, 0.8329, and 0.3741 in Stage 2 [40]. The results indicated a considerable variation in efficiency scores across provinces, such that in the adjusted model, provinces like Semnan (0.54), Tehran (0.54), and Golestan (0.55) demonstrated higher levels of efficiency, while provinces such as South Khorasan (0.42), Alborz (0.43), and Kerman (0.43) showed lower values. This inter-provincial variation, along with intra-provincial heterogeneity, may reflect the significant role of managerial differences, infrastructural capacity, and resource allocation in shaping hospital performance during the COVID-19 crisis. Such differences in the efficiency of various subsectors of Iran’s health system across regions are not limited to COVID-19. Similar patterns have also been reported in studies evaluating the overall efficiency of hospitals under non-pandemic conditions, as well as in areas such as primary care systems. For instance, Shaker et al., using Data Envelopment Analysis (DEA), evaluated the technical efficiency of financial and human resources in public hospitals across 31 provinces. Efficiency scores varied widely: East Azerbaijan, Razavi Khorasan, and Gilan reached full efficiency [1], while Hamadan and Kerman scored below 0.3 [41]. Iran has been under severe economic sanctions at the same time as the pandemic, a situation that compromised Iran’s health system. Although medicines and medical equipment are exempted from the economic sanctions, the direct and indirect effects of sanctions have restricted Iran’s banking system and consequently have led to a wide range of limitations on trade, manufacturing sector, insurance, and ventures [42]. This situation increases the need to adopt appropriate strategies to improve efficiency.

On average, about 42% of each patient’s bill costs were the costs of medicine and medical consumables, so this group can be considered the most important driver of costs. Koushk et al. in a study in a systematic review have categorized various strategies and actions proposed in the literature for in-hospital drug cost containment into 5 groups: Procurement, Storage, Distribution, Prescription, and Use. Some measures related to prescription and use are prevention from duplication prevention, Substitution of less expensive alternatives, Rules and regulations, prescription of generic drugs, regularly ranking and limiting the use of top-ranked drugs, A computerized decision-support program connected to computer-based patient records, Physician education, The rounding of drug dosages, Returning patients surplus drugs and deliver to hospital pharmacy [43].

The second group with the highest bill shares was hoteling services (charges for hospital beds and nursing care in intensive/non-intensive care units). On average, this group accounts for about 29% of the bill. Increasing the length of stay in the hospital and being cared for in intensive care units increases the share of this group in the bill. Several studies have provided estimates of the length of stay (LOS) for patients with COVID-19. For example, on average, 4.8 days to 8 days in different provinces of Iran [44], and 4.94 days in Hormozgan Province of Iran [45]. In the systematic review and meta-analysis conducted by Mohammadi et al., the mean LOS was reported to be 15.35 days [46]. One of the reasons for increasing the hoteling charges in the bill can be being under care in the ICU, which is one of the relatively expensive services in health systems [47]. For example, in Iran, the per-day charge for ICU care at a public hospital in 2021 was 12,159,000 IRR, while it was 2,620,000 IRR for per-day non-ICU care simultaneously [48]. Evidence from different settings has shown that many hospital stays were unnecessary and inappropriate. Moradi et al. in a study in a specialized burn hospital, reported that about 28.5% of the patients had at least 1 day of inappropriate stay, and about 6% of the total hospitalization days were inappropriate [49]. In a systematic review and meta-analysis, Arab-Zozani et al. showed that in Iran, the overall rate of inappropriate ICU admissions was 12.3% [50]. Developing clinical guidelines, and their education and providing appropriate motivation to follow them in clinical environments can be effective strategies to reduce clinical practice variations, clinical care quality assurance, and improve efficiency. Clinical guidelines are “statements that include recommendations intended to optimize patient care that is informed by a systematic review of evidence and an assessment of the benefits and harms of alternative care options”. They have the potential to reduce unwarranted practice variation, enhance the translation of research into practice, and improve healthcare quality and safety if developed and implemented according to international standards [51]. The evidence shows that paper-based clinical guidelines are used less in practice, and the development of Clinical Decision Support Systems (CDSS) can significantly contribute to the up-to-date use of international clinical evidence gathered in the form of clinical guidelines [52].

This study has several strengths. The main strength of this study is its dataset. It includes more than 439,000 hospital records from 493 public hospitals across the country. Having such a large and diverse dataset gives a solid base for analysis and makes the results more reliable and applicable. Second, the methodological framework is innovative: by combining data envelopment analysis (DEA) with risk-adjusted survival measures and complementary regression approaches, the study offers a more refined understanding of hospital efficiency. Third, looking at the data from the perspective of the national health insurer offers useful insights for policy, especially regarding resource allocation and financial planning during crises. At the same time, some limitations need to be noted. The DEA model relied on a relatively small set of inputs and outputs, which means it may not fully reflect all aspects of hospital performance. To address this, we included risk-adjusted survival measures and variables related to epidemic waves, helping to account for differences between patients and changes over time. Another challenge comes from the diversity among hospitals. Differences in size, teaching status, and available resources can make direct comparisons difficult. We partly addressed this by using regression analyses to adjust for these contextual factors. A further limitation of this study is the absence of detailed clinical information, such as specific comorbidities or laboratory findings, which would have enabled a more precise adjustment for patient case-mix differences. To partly address this gap, patient age and ICU admission were used as proxy indicators of disease severity, and risk-adjusted survival was incorporated as the output in the DEA model to account, as far as possible, for underlying variations in clinical complexity across hospitals. These measures helped reduce, though not eliminate, the potential bias arising from differences in patient complexity across hospitals. Finally, although data from 2020 were available, our analysis focused on 2021. This was done to avoid distortions caused by the health system’s initial unpreparedness during the early months of the pandemic. By 2021, hospitals were assumed to be operating under more stable conditions.

Future research should build on this work by incorporating richer clinical and structural indicators including comorbidities, treatment modalities, and hospital resource measures to provide a more comprehensive picture of efficiency determinants. In addition, a deeper exploration of temporal dynamics, such as the impact of different pandemic waves, resource shortages, and workforce pressures, would offer further insights into efficiency variations under crisis conditions. Such efforts can help inform evidence-based and equitable policies aimed at strengthening the resilience of health systems in the face of future public health emergencies.

## Conclusion

This study is the first nationwide effort in Iran to evaluate how efficiently public hospitals managed patients hospitalized with COVID-19, using routinely collected administrative data. The findings revealed wide differences in daily hospital charges and indicated that, on average, hospitals operated below their optimal efficiency levels. These results suggest that there remains meaningful potential to improve the use of available resources through well-designed policy and management reforms. Drawing on these findings, several directions can guide policymakers and health system managers:

- Strengthen adherence to clinical guidelines to reduce unwarranted variations in care and enhance consistency across hospitals.
- Expand the use of decision-support and monitoring tools that enable data-driven management and timely performance feedback.
- Adopt targeted charge-control and budgeting strategies to improve cost containment while safeguarding patient outcomes.
- Encourage evidence-based efficiency benchmarking across hospitals to identify best practices and guide capacity adjustments.

Despite certain limitations, this study captures an overall picture of hospital performance and spending patterns across Iran during the COVID-19 pandemic. The insights gained can support policymakers in designing strategies that enhance the system’s financial sustainability and operational readiness for future health challenges. Finally, this study makes a unique contribution by combining a payer-perspective DEA approach with risk-adjusted survival as an output indicator. This analytical design has not been previously applied to hospital efficiency evaluation in Iran.

## Supplementary Information

Supplementary Material 1.

Supplementary Material 2.

Supplementary Material 3.

## Data Availability

The datasets used and/or analyzed during the current study are available from the corresponding author upon reasonable request.

## References

[1] Zeng W, Yao Y, Barroy H, Cylus J, Li G. Improving fiscal space for health from the perspective of efficiency in low-and middle-income countries: what is the evidence? J Glob Health. 2020. 10.7189/jogh.10.020421. 33110580 10.7189/jogh.10.020421 PMC7568933

[2] Khatri N. Crony Capitalism in US Health Care: Anatomy of a Dysfunctional System. Routledge; 2021.

[3] Aboulhallaje M, Najafi B, Kia Daliri AAA. Measuring the technical efficiency of Iranian ministry of health and medical education hospitals: 2007. Teb Va Tazkieh. 2010;19(3):49–61.

[4] Salehzadeh R, Ketabi S. Measuring the efficiency of Qom hospitals with data envelopment analysis and analytic hierachy process. Health Inform Manage. 2011;8(4):489.

[5] Jabbari A, Jafarian M, Khorasani E, Ghaffari M, Majlesi M. Emergency department waiting time at Alzahra hospital. Director Gen. 2011;8(4):500–11.

[6] World Health Organization. Tracking health expenditure on COVID-19 within the system of health accounts framework: technical note, June 2022. World Health Organization; 2022.

[7] Li K, Al-Amin M, Rosko MD. Early financial impact of the COVID-19 pandemic on US hospitals. J Healthc Manag. 2023;68(4):268–83. 37410989 10.1097/JHM-D-22-00175 PMC10306278

[8] Kaye AD, Okeagu CN, Pham AD, Silva RA, Hurley JJ, Arron BL, et al. Economic impact of COVID-19 pandemic on healthcare facilities and systems: international perspectives. Best Pract Res Clin Anaesthesiol. 2021;35(3):293–306. 34511220 10.1016/j.bpa.2020.11.009 PMC7670225

[9] World Health Organization. Spending on health in Europe: entering a new era Copenhagen: WHO Regional Office for Europe. 2021. Available from: https://iris.who.int/bitstream/handle/10665/340910/9789289055079-eng.pdf?sequence=1&isAllowed=y.

[10] Su EC-Y, Hsiao C-H, Chen Y-T, Yu S-H, editors. An examination of COVID-19 mitigation efficiency among 23 countries. Healthcare: MDPI; 2021. 10.3390/healthcare9060755 PMC8235777 34207404

[11] Selamzade F, Ersoy Y, Ozdemir Y, Celik MY. Health efficiency measurement of OECD countries against the COVID-19 pandemic by using DEA and MCDM methods. Arab J Sci Eng. 2023;48(11):15695–712.

[12] Kuzior A, Kashcha M, Kuzmenko O, Lyeonov S, Brożek P. Public health system economic efficiency and COVID-19 resilience: frontier DEA analysis. Int J Environ Res Public Health. 2022;19(22):14727. 36429444 10.3390/ijerph192214727 PMC9690233

[13] Breitenbach MC, Ngobeni V, Aye GC. Global healthcare resource efficiency in the management of COVID-19 death and infection prevalence rates. Front Public Health. 2021;9:638481. 33996718 10.3389/fpubh.2021.638481 PMC8116650

[14] Henriques C, Gouveia M. Assessing the impact of COVID-19 on the efficiency of Portuguese state-owned enterprise hospitals. Socio-Econ Plann Sci. 2022;84:101387. 10.1016/j.seps.2022.101387 PMC9339160 35937707

[15] Pecoraro F, Luzi D, Clemente F. The efficiency in the ordinary hospital bed management: a comparative analysis in four European countries before the COVID-19 outbreak. PLoS ONE. 2021;16(3):e0248867. 33750956 10.1371/journal.pone.0248867 PMC7984624

[16] Mishra V, Singh J, Kulkarni S, Yadav S. Analysis of profit efficiency of corporate hospitals in India during COVID-19–an DEA-MPI based approach. Int J Healthc Manag. 2024;17(1):177–85.

[17] Sülkü SN, Mortaş A, Küçük A. Measuring efficiency of public hospitals under the impact of Covid-19: the case of Türkiye. Cost Eff Resource Allocation. 2023;21(1):1–12. 10.1186/s12962-023-00480-6 PMC10521413 37749589

[18] United Nations. UN Iran socio-economic recovery programme against the impact of. COVID-19. 2020. Available from: https://www.undp.org/sites/g/files/zskgke326/files/migration/ir/ed815d9fcb36963c575d3c16b09163564da1afd8e960cdbaa4c0d08a5d8ad3b2.pdf.

[19] Damiri S, Goharimehr M, Nasehi MM, Effatpanah M, Shahali Z, Ranjbaran H, et al. COVID-19 burden in Iran: disability-adjusted life years analysis from hospital data, 2020–2021. Arch Public Health. 2024;82(1):135. 39187892 10.1186/s13690-024-01355-9 PMC11346186

[20] Ghaffari Darab M, Keshavarz K, Sadeghi E, Shahmohamadi J, Kavosi Z. The economic burden of coronavirus disease 2019 (COVID-19): evidence from Iran. BMC Health Serv Res. 2021;21:1–7. 33573650 10.1186/s12913-021-06126-8 PMC7877330

[21] Damiri S, Nahvijou A, Sargazi N, Fazaeli AA, Akbari Sari A, Daroudi R. Hospitalization costs of patients with Covid-19: a study in Tehran university of medical sciences. Health Manage Inform Sci. 2021;8(3):168–76.

[22] Mirhashemi SH, Mostafavi H, Mollajafari F, Ahmad ZZ, Hashempour R. Direct medical cost and cost analysis of COVID-19 in Iran: a multicenter cross-sectional study. Int J Crit Illn Inj Sci. 2022;12(1):10–6. 35433401 10.4103/ijciis.ijciis_57_21 PMC9008290

[23] Karimi F, Ezzati F, Sadeghifar J, Bazyar M, Dargahpour M. Direct medical costs analysis of Covid-19 patients in the hospitals of Ilam university of medical sciences. Evidence Based Health Policy, Management and Economics. 2022.

[24] Statistical Centre of Iran. The statistical yearbook of Iran 2021–2022: health and treatment. Iran: Tehran; 2021.

[25] Mirmozaffari M, Yazdani R, Shadkam E, Khalili SM, Tavassoli LS, Boskabadi A. A novel hybrid parametric and non-parametric optimisation model for average technical efficiency assessment in public hospitals during and post-COVID-19 pandemic. Bioengineering. 2021;9(1):7. 35049716 10.3390/bioengineering9010007 PMC8772782

[26] Zacharia PK. Operational factors related to performance of health facilities in implementing National health insurance fund online claims management information system. Muhimbili University of Health and Allied Sciences. 2021.

[27] ICD-10 Version. 2019. International Statistical Classification of Diseases and Related Health Problems 10th Revision (ICD-10)-WHO Version for;2019-covid-expanded. 2024. Available from: https://icd.who.int/browse10/2019/en#/U07.1.

[28] Charnes A, Cooper WW, Rhodes E. Measuring the efficiency of decision making units. Eur J Oper Res. 1978;2(6):429–44.

[29] Banker RD, Charnes A, Cooper WW. Some models for estimating technical and scale inefficiencies in data envelopment analysis. Manage Sci. 1984;30(9):1078–92.

[30] Clement JP, Valdmanis VG, Bazzoli GJ, Zhao M, Chukmaitov A. Is more better? An analysis of hospital outcomes and efficiency with a DEA model of output congestion. Health Care Manag Sci. 2008;11(1):67–77. 18390169 10.1007/s10729-007-9025-8

[31] Bilsel M, Davutyan N. Hospital efficiency with risk adjusted mortality as undesirable output: the Turkish case. Ann Oper Res. 2014;221(1):73–88.

[32] Ayala A, Villalobos Dintrans P, Elorrieta F, Castillo C, Vargas C, Maddaleno M. Identification of COVID-19 waves: considerations for research and policy. Int J Environ Res Public Health. 2021. 10.3390/ijerph182111058. 34769577 10.3390/ijerph182111058 PMC8583384

[33] Harvey J, Chan B, Srivastava T, Zarebski AE, Dłotko P, Błaszczyk P, et al. Epidemiological waves - Types, drivers and modulators in the COVID-19 pandemic. Heliyon. 2023;9(5):e16015. 37197148 10.1016/j.heliyon.2023.e16015 PMC10154246

[34] Malekzadeh R, Tavana M, Abedi G, Ziapour A, Abedini E. Comparing the efficiency of hospitals in Northern Iran before and after the Covid-19 pandemic using the Pabon Lasso model. Afr J Nurs Midwifery. 2023;25(2).

[35] Raadabadi M, Tolideh Z, Shoara Z, Yeganeh Z, Sadeghifar J, Momeni K. Comparing the efficiency of hospitals in Western Iran before and after the Covid-19 pandemic using the Pabon Lasso model. Payavard Salamat. 2024;18(1):44–52.

[36] Mahmoodi A, Taji M, Ayask FA. Evaluating the performance of inpatient wards in hospitals affiliated with Birjand university of medical sciences using the Pabon-Lasso model in the years before and during the COVID-19 pandemic. Q J Manage Strategies Health Syst. 2023.

[37] Khandehroo M, Dorri M, Paykani T, Khajavi A, Joshani-Kheibari M, Esmaeili R. Direct inpatient cost and payments of COVID-19 in Iran: quantile regression analysis. Med J Islam Repub Iran. 2022;36:101. 36447539 10.47176/mjiri.36.101 PMC9700402

[38] Ramezani-Doroh V, Tapak L, Hamidi Y, Bashirian S, Soltanian AR, Motaghed M, et al. Which patients bring the most costs for hospital? A study on the cost determinants among COVID-19 patients in Iran. Cost Eff Resour Alloc. 2022;20(1):52. 36153533 10.1186/s12962-022-00386-9 PMC9509555

[39] Aghajani MH, Sistanizad M, Toloui A, Neishaboori AM, Pourhoseingholi A, Maher A, et al. COVID-19 related hospitalization costs; assessment of influencing factors. Front Emerg Med. 2022;6(1):e3–e.

[40] Babaei-Meybodi H, Moradi H, Abbaszadeh M. Evaluation of the proficiency of distinguished countries in managing COVID-19. Health Inform Manage. 2021;18(1):19–26. [in persian].

[41] SHaker Z, SHaker Z, Barouni M, Sabermahany A. Financial and human resources distribution efficiency of public hospitals by provinces in Iran 2018. Hospital. 2022;21(1):44–51.

[42] Abdoli A. Iran, sanctions, and the COVID-19 crisis. J Med Econ. 2020;23(12):1461–5. 33249954 10.1080/13696998.2020.1856855

[43] Koushki MS, Goudarzi R, Amiresmaili M, Nekooei Moghaddam M, Yazdi-Feyzabadi V, Talebian A. Strategies of drugs cost containment in hospital: a systematic literature review. Int J Health Plann Manag. 2023;38(1):7–21. 10.1002/hpm.3542 36100961

[44] Damiri S, Shojaee A, Dehghani M, Shahali Z, Abbasi S, Daroudi R. National geographical pattern of COVID-19 hospitalization, case fatalities, and associated factors in patients covered by Iran health insurance organization. BMC Public Health. 2022;22(1):1274. 35773657 10.1186/s12889-022-13649-0 PMC9243909

[45] Mastaneh Z, Mouseli A, Mohseni S, Dadipoor S. Predictors of hospital length of stay and mortality among COVID-19 inpatients during 2020–2021 in Hormozgan Province of Iran: a retrospective cohort study. Health Sci Rep. 2023;6(6):e1329. 37324249 10.1002/hsr2.1329 PMC10265171

[46] Alimohamadi Y, Yekta EM, Sepandi M, Sharafoddin M, Arshadi M, Hesari E. Hospital length of stay for COVID-19 patients: a systematic review and meta-analysis. Multidisciplinary Respiratory Med. 2022;17(1). 10.4081/mrm.2022.856 PMC9472334 36117876

[47] Chacko B, Ramakrishnan N, Peter JV. Approach to intensive care costing and provision of cost-effective care. Indian J Crit Care Med. 2023;27(12):876–87. 38074956 10.5005/jp-journals-10071-24576 PMC10701560

[48] Tariff for diagnostic and treatment services for the government health service delivery sector. 2021.

[49] Moradi N, Moradi K, Bagherzadeh R, Aryankhesal A. Rate and causes of inappropriate stays and the resulting financial burden in a single specialty burns hospital. BMC Health Serv Res. 2022;22(1):1538. 36527082 10.1186/s12913-022-08772-y PMC9758030

[50] Arab-Zozani M, Pezeshki MZ, Khodayari-Zarnaq R, Janati A. Inappropriate rate of admission and hospitalization in the Iranian hospitals: a systematic review and meta-analysis. Value Health Reg Issues. 2020;21:105–12. 31704488 10.1016/j.vhri.2019.07.011

[51] World Health Organization. Clinical Practice Guidelines as a quality strategy. Improving Healthcare Quality in Europe Characteristics, Effectiveness and Implementation of Different Strategies: Characteristics, Effectiveness and Implementation of Different Strategies. Copenhagen (Denmark). 2019.

[52] Kilsdonk E, Peute L, Jaspers MW. Factors influencing implementation success of guideline-based clinical decision support systems: a systematic review and gaps analysis. Int J Med Inform. 2017;98:56–64. 28034413 10.1016/j.ijmedinf.2016.12.001

