# Supplementary material for: Efficiency in COVID-19 inpatient care: findings from public hospitals in Iran
Source: Health Econ Rev. 2025 Nov 24;15:101. doi: 10.1186/s13561-025-00696-7 (PMC12642060; doi:10.1186/s13561-025-00696-7)
Supplement: Supplementary file 1 — Supplementary Material 1. [file 13561_2025_696_MOESM1_ESM.docx]

Table S1. Summary statistics of COVID-19 hospitalized patients and hospital outcomes across Iranian provinces

| Province | hospitals (N) | Inpatients (N) | Age | | Outcomes | | Length of hospital stay | | | | | | |
| --- | --- | --- | --- | --- | --- | --- | --- | --- | --- | --- | --- | --- | --- |
|  |  |  |  |  | ICU admission rate (%) | Mortality rate (%) | Total days of hospital stay (N) | The length of each patient's hospital stays | | Average ICU length of stay | | The proportion of ICU stay from total days of hospital stay | |
|  |  |  | Mean | std-error |  |  |  | Mean | std-error | Mean | std-error | Mean | std-error |
| East Azerbaijan | 28 | 20,882 | 57.85 | 0.14 | 18.4 | 10.9 | 126,442 | 6.06 | 0.04 | 1.18 | 0.02 | 13.50 | 0.21 |
| West Azerbaijan | 25 | 28,037 | 53.87 | 0.12 | 16.8 | 7.9 | 151,003 | 5.39 | 0.02 | 0.92 | 0.02 | 11.68 | 0.17 |
| Ardabil | 9 | 9,228 | 56.85 | 0.21 | 11.5 | 8.2 | 49,874 | 5.40 | 0.05 | 0.75 | 0.03 | 7.51 | 0.24 |
| Isfahan | 33 | 27,640 | 60.41 | 0.12 | 16.4 | 11.0 | 176,130 | 6.37 | 0.03 | 1.04 | 0.02 | 10.01 | 0.15 |
| Alborz | 10 | 8,014 | 55.56 | 0.22 | 20.1 | 14.0 | 46,194 | 5.76 | 0.05 | 1.09 | 0.04 | 13.33 | 0.33 |
| Ilam | 8 | 6,132 | 53.34 | 0.26 | 18.6 | 4.5 | 25,405 | 4.14 | 0.04 | 0.68 | 0.03 | 10.97 | 0.33 |
| Bushehr | 8 | 4,634 | 52.95 | 0.28 | 9.4 | 7.9 | 22,967 | 4.96 | 0.06 | 0.51 | 0.03 | 5.96 | 0.30 |
| Tehran | 44 | 32,816 | 54.39 | 0.12 | 29.3 | 13.7 | 218,782 | 6.67 | 0.03 | 1.86 | 0.02 | 20.81 | 0.20 |
| ChaharM & Bakhtiari | 7 | 7,608 | 54.28 | 0.28 | 11.7 | 7.0 | 40,737 | 5.35 | 0.05 | 0.66 | 0.03 | 6.95 | 0.25 |
| South Khorasan | 10 | 8,385 | 57.11 | 0.26 | 12.3 | 5.5 | 35,082 | 4.18 | 0.04 | 0.55 | 0.03 | 8.23 | 0.26 |
| Razavi Khorasan | 26 | 33,344 | 56.83 | 0.13 | 19.0 | 15.0 | 190,143 | 5.70 | 0.03 | 1.08 | 0.02 | 12.85 | 0.16 |
| North Khorasan | 9 | 10,242 | 53.76 | 0.20 | 10.5 | 6.0 | 47,912 | 4.68 | 0.04 | 0.51 | 0.02 | 6.64 | 0.21 |
| Khuzestan | 24 | 22,151 | 50.95 | 0.15 | 22.2 | 11.3 | 117,698 | 5.31 | 0.03 | 1.35 | 0.03 | 15.80 | 0.22 |
| Zanjan | 7 | 6,945 | 58.01 | 0.25 | 15.0 | 8.4 | 39,674 | 5.71 | 0.05 | 0.83 | 0.03 | 9.77 | 0.31 |
| Semnan | 5 | 4,276 | 58.45 | 0.29 | 22.6 | 8.3 | 23,938 | 5.60 | 0.06 | 1.31 | 0.05 | 16.79 | 0.51 |
| Sistan & Baluchestan | 11 | 7,239 | 50.23 | 0.26 | 17.3 | 13.3 | 32,611 | 4.50 | 0.05 | 0.79 | 0.03 | 12.66 | 0.36 |
| Fars | 32 | 26,454 | 55.24 | 0.13 | 12.4 | 8.4 | 135,170 | 5.11 | 0.03 | 0.68 | 0.02 | 8.72 | 0.15 |
| Qazvin | 8 | 6,517 | 52.93 | 0.29 | 18.4 | 9.6 | 36,608 | 5.62 | 0.06 | 1.11 | 0.04 | 11.00 | 0.32 |
| Qom | 6 | 6,745 | 49.58 | 0.33 | 10.7 | 12.5 | 40,364 | 5.98 | 0.06 | 0.61 | 0.03 | 6.54 | 0.26 |
| Kurdistan | 13 | 12,969 | 56.20 | 0.17 | 10.0 | 6.0 | 67,151 | 5.18 | 0.03 | 0.56 | 0.02 | 6.32 | 0.18 |
| Kerman | 21 | 23,689 | 51.86 | 0.15 | 11.8 | 7.3 | 120,950 | 5.11 | 0.03 | 0.71 | 0.02 | 7.44 | 0.15 |
| Kermanshah | 17 | 13,986 | 53.86 | 0.17 | 13.3 | 6.5 | 73,560 | 5.26 | 0.03 | 0.76 | 0.02 | 9.94 | 0.23 |
| Kohgiluyeh & BoyerA | 5 | 6,781 | 52.25 | 0.26 | 8.6 | 3.7 | 36,091 | 5.32 | 0.05 | 0.54 | 0.03 | 5.04 | 0.22 |
| Golestan | 16 | 11,755 | 51.03 | 0.21 | 14.5 | 10.9 | 69,752 | 5.93 | 0.04 | 0.84 | 0.03 | 9.18 | 0.23 |
| Gilan | 19 | 10,181 | 56.11 | 0.21 | 9.9 | 10.7 | 53,116 | 5.22 | 0.04 | 0.55 | 0.02 | 6.33 | 0.21 |
| Lorestan | 14 | 17,685 | 52.33 | 0.17 | 11.0 | 5.3 | 85,483 | 4.83 | 0.03 | 0.55 | 0.02 | 7.39 | 0.17 |
| Mazandaran | 26 | 18,846 | 54.85 | 0.16 | 16.7 | 6.8 | 105,325 | 5.59 | 0.03 | 0.91 | 0.02 | 12.65 | 0.22 |
| Markazi | 14 | 6,680 | 61.25 | 0.25 | 13.6 | 8.1 | 35,699 | 5.34 | 0.05 | 0.77 | 0.03 | 8.83 | 0.30 |
| Hormozgan | 13 | 13,799 | 47.69 | 0.19 | 13.1 | 5.7 | 59,635 | 4.32 | 0.03 | 0.65 | 0.02 | 8.63 | 0.21 |
| Hamadan | 15 | 18,635 | 55.65 | 0.15 | 14.2 | 5.9 | 104,144 | 5.59 | 0.03 | 0.78 | 0.02 | 9.52 | 0.19 |
| Yazd | 10 | 6,884 | 57.41 | 0.26 | 13.9 | 6.2 | 32,857 | 4.77 | 0.04 | 0.76 | 0.03 | 9.44 | 0.31 |
| Total | 493 | 439,179 | 55 | 0 | 16.0 | 9.2 | 2,400,497 | 5.46 | 0.01 | 0.91 | 0.00 | 10.88 | 0.04 |
