# Supplementary material for: Efficiency in COVID-19 inpatient care: findings from public hospitals in Iran
Source: Health Econ Rev. 2025 Nov 24;15:101. doi: 10.1186/s13561-025-00696-7 (PMC12642060; doi:10.1186/s13561-025-00696-7)
Supplement: Supplementary file 2 — Supplementary Material 2. [file 13561_2025_696_MOESM2_ESM.docx]

**Table S2.** Results of logistic regression analysis for risk-adjusted survival

| **Variable** | **Groups** | **Coefficient (β)** | **Std. Error** | **Odds Ratio (OR=exp(β))** | **95% CI for OR** | **p-value** |
| --- | --- | --- | --- | --- | --- | --- |
| **Wave** | **Ref = non-wave** | | | | | |
|  | Wave 1 (2021/03/21-2021/06/08) | -0.065 | 0.015 | 0.94 | 0.91 – 0.97 | <0.001 |
|  | Wave 2 (2021/06/08-2021/08/21) | 0.135 | 0.016 | 1.14 | 1.11 – 1.18 | <0.001 |
|  | Wave 3 (2022/01/17-2022/03/20) | -0.289 | 0.022 | 0.75 | 0.72 – 0.78 | <0.001 |
| **Age groups** | **Ref = <20** | | | | | |
|  | Age group 1(21-50) | 0.649 | 0.043 | 1.91 | 1.76 – 2.08 | <0.001 |
|  | Age group 2(>50) | 1.869 | 0.04 | 6.48 | 5.99 – 7.01 | <0.001 |
| **Gender** | **Ref=Male** | | | | | |
|  | Female | -0.238 | 0.012 | 0.79 | 0.77 – 0.81 | <0.001 |
| **ICU admission** | **Ref=Not admitted to ICU** | | | | | |
|  | Admitted to ICU | 2.715 | 0.012 | 15.1 | 14.8 – 15.5 | <0.001 |
| **Constant** | | -4.647 | 0.041 | 0.01 | – | <0.001 |
| Model fit statistics  Number of observations: 435,964  Log likelihood: –99,256.99  LR χ²(7): 67,690.75, p<0.001  Pseudo R²: 0.254 | | | | | | |
