# Supplementary material for: Efficiency in COVID-19 inpatient care: findings from public hospitals in Iran
Source: Health Econ Rev. 2025 Nov 24;15:101. doi: 10.1186/s13561-025-00696-7 (PMC12642060; doi:10.1186/s13561-025-00696-7)
Supplement: Supplementary file 3 — Supplementary Material 3. [file 13561_2025_696_MOESM3_ESM.docx]

**Table S3.**  Determinants of hospital efficiency: Fractional logit model

| **Variable** | **Coefficient (β)** | **Std. Error** | **p-value** | **95% CI (β)** | **Marginal effect** | **Std. Error** | **p-value** | **95% CI** |
| --- | --- | --- | --- | --- | --- | --- | --- | --- |
| Teaching hospital (Ref = non-teaching) | 0.101 | 0.124 | 0.417 | -0.143 – 0.344 | 0.024 | 0.03 | 0.415 | -0.034 – 0.083 |
| Single-specialty (Ref = General) | -0.339 | 0.173 | 0.05 | -0.679 – 0.001 | -0.084 | 0.043 | 0.053 | -0.169 – 0.001 |
| ln (Beds) | 0.158 | 0.065 | 0.016 | 0.030 – 0.285 | 0.038 | 0.016 | 0.015 | 0.007 – 0.069 |
| Province (Ref = East Azerbaijan) ^*^ | | | | | | | | |
| West Azerbaijan | 0.646 | 0.293 | 0.028 | 0.071 – 1.220 | 0.15 | 0.067 | 0.025 | 0.019 – 0.281 |
| Tehran | -1.088 | 0.228 | <0.001 | -1.535 – -0.640 | -0.259 | 0.055 | <0.001 | -0.366 – -0.151 |
| North Khorasan | 1.069 | 0.476 | 0.025 | 0.137 – 2.002 | 0.23 | 0.089 | 0.009 | 0.057 – 0.404 |
| Semnan | -0.637 | 0.209 | 0.002 | -1.047 – -0.227 | -0.157 | 0.052 | 0.002 | -0.259 – -0.056 |
| Kerman | 0.924 | 0.439 | 0.035 | 0.064 – 1.784 | 0.204 | 0.087 | 0.019 | 0.033 – 0.375 |
| Golestan | -0.447 | 0.243 | 0.066 | -0.924 – 0.029 | -0.111 | 0.06 | 0.064 | -0.229 – 0.006 |
| Lorestan | 1.162 | 0.352 | 0.001 | 0.473 – 1.851 | 0.246 | 0.068 | <0.001 | 0.112 – 0.379 |
| Mazandaran | -0.424 | 0.245 | 0.083 | -0.903 – 0.055 | -0.106 | 0.06 | 0.081 | -0.224 – 0.013 |
| Hormozgān | 0.717 | 0.265 | 0.007 | 0.197 – 1.237 | 0.164 | 0.061 | 0.007 | 0.045 – 0.283 |
| Constant | -0.631 | 0.353 | 0.074 | -1.324 – 0.061 |  | | | |
| Model fit statistics  Number of observations: 493  Wald χ²(33) = 335.76, p<0.001  Pseudo R² = 0.058  Log pseudolikelihood = -281781.54 | | | | | | | | |

*Only provinces with statistically significant coefficients are presented in this table
